# Antiangiogenic Activity of 6-O-Desulfated Modified Heparin: Suppression of Choroidal Neovascularization

**DOI:** 10.3390/ijms26167673

**Published:** 2025-08-08

**Authors:** Alex Treiger Grupenmacher, Bianca Oliveira Augusto, Bruna Zancanelli Fetter, Juliana P. Rocha, Diego Lisboa Araujo, Vinicius Kniggendorf, Helena B. Nader, Caio Vinicius Saito Regatieri, Juliana L. Dreyfuss

**Affiliations:** 1 Department of Ophthalmology and Visual Sciences, Escola Paulista de Medicina, Universidade Federal de São Paulo, São Paulo 04023-062, Brazil; alexgrups@gmail.com (A.T.G.); julianapimentelr@gmail.com (J.P.R.); diego_lisboa10@hotmail.com (D.L.A.); vinicius_kdorf@yahoo.com.br (V.K.); regatieri@unifesp.br (C.V.S.R.); 2 Department of Biochemistry, Molecular Biology Division, Escola Paulista de Medicina, Universidade Federal de São Paulo, São Paulo 04044-020, Brazil; biancaoliveiraugusto@gmail.com (B.O.A.); bruzfetter@gmail.com (B.Z.F.); hbnader@unifesp.br (H.B.N.)

**Keywords:** age-related macular degeneration, angiogenesis, antiangiogenesis, heparin, choroidal neovascularization, endothelial cells, FGF-2, intravitreal injection

## Abstract

Age-related macular degeneration (AMD) is a leading cause of irreversible blindness worldwide, primarily due to pathological choroidal neovascularization (CNV). Our study investigates a chemically modified heparin derivative as a novel strategy to selectively modulate angiogenic signaling, offering a reduced anticoagulant risk and preclinical support for AMD treatment. We explored the therapeutic potential of 6-O-desulfated heparin (Hep-6Od) as an antiangiogenic agent with diminished anticoagulant activity. Synthesized via selective 6-O-desulfation and characterized using nuclear magnetic resonance (NMR), Hep-6Od demonstrated safety in retinal pigment epithelial cells with no cytotoxic effects at various concentrations. In vitro, the compound significantly inhibited endothelial cell proliferation, migration, and capillary tube formation. Differential scanning fluorimetry (DSF) assays confirmed molecular interaction between Hep-6Od and fibroblast growth factor 2 (FGF-2), suggesting interference with pro-angiogenic signaling pathways. In vivo, a laser-induced CNV model in lean Zucker rats showed a dose-dependent reduction in neovascular lesion areas after an intravitreal Hep-6Od injection. Compared to unfractionated heparin, Hep-6Od exhibited reduced anticoagulant effects in PT and aPTT assays while maintaining robust antiangiogenic properties. These findings support Hep-6Od as a promising alternative to anti-vascular endothelial growth factor (VEGF) therapies for AMD treatment, potentially expanding current retinal vascular disease interventions. The results underscore its potential to transform AMD management, pending further clinical validation and awaiting confirmation in further studies.

## 1. Introduction

Age-related macular degeneration (AMD) is the major cause of irreversible blindness in developed countries among individuals aged 50 and older [1], affecting 8.7% of the world population, with estimates reaching over 288 million people by 2040 [2]. AMD manifests in two forms, dry (atrophic) and wet (neovascular or exudative), with the milestone of the latter being choroidal neovascularization, the formation of new blood vessels in the choroid, a layer of tissue beneath the retina. These new vessels can leak fluid and blood, leading to scarring and central vision loss.

Choroidal neovascularization in AMD is driven by basal lamina and basal linear deposits of cellular debris between the retina pigment epithelium (RPE) and Bruch’s membrane [3,4]. In wet AMD, inflammatory factors are overexpressed, as hypoxia inducible factor 1 (HIF-1) triggers the release of several pro-angiogenic factors, notably vascular endothelial growth factor A (VEGF-A), alongside angiopoietin 2, platelet-derived growth factor (PDGF), and fibroblast growth factor 2 (FGF2) [5]. When up-regulated, these molecules start complex processes of extracellular matrix remodeling through interactions with local molecules, such as glycosaminoglycans (GAGs), facilitating vascular sprouting, cellular migration, and tube formation through processes known as angiogenesis and vasculogenesis [6]. Without intervention, these processes can lead to fibrosis, scarring, and ultimately, irreversible vision loss [5,7].

Among the variety of sulfated glycosaminoglycans (GAGs), heparin and heparan sulfate (HS) are particularly recognized for their pivotal roles in extracellular matrix remodeling during angiogenesis and inflammation [8,9]. The biological roles of these GAGs extend beyond anticoagulation to critically regulate angiogenesis, cell proliferation, migration, and inflammatory responses. While heparin is predominantly stored in mast cell granules, HS proteoglycans are ubiquitously expressed at the cell surface and in the extracellular matrix (ECM), where they modulate key signaling pathways [9,10]. These effects are mediated by the ability of their structurally diverse polysaccharide chains to interact selectively with a range of signaling proteins. Specific sulfation motifs and conformational domains within HS chains enable binding to angiogenic regulators such as VEGF, FGF-2, HGF, PDGF-B, TGF-β, thrombospondin-1 (TSP-1), platelet factor 4 (PF4), and endostatin, as well as to inflammatory cytokines, chemokines, morphogens, and enzymes [10,11,12]. Notably, VEGF165, the most clinically relevant isoform, contains a defined heparin/HS-binding domain that is critical for its bioavailability, receptor engagement, and angiogenic activity [8,11]. A key example is FGF-2, whose mitogenic and pro-angiogenic signaling depend on HS acting as a co-receptor: HS stabilizes the FGF-FGFR complex, promoting receptor dimerization and activation of the MAPK and PI3K/Akt pathways, with 6-O- and N-sulfation playing essential roles in binding specificity and signaling efficacy [10,11,13,14]. These cascades drive endothelial cell proliferation, migration, and cytoskeletal remodeling. In inflammatory conditions, HS modulates chemokine gradients and leukocyte adhesion, further contributing to vascular remodeling. Altogether, the multifaceted regulatory functions of heparin and HS underscore their importance in the molecular control of pathological neovascularization and inflammation. These multifaceted roles highlight the importance of heparin and HS in coordinating the molecular events underlying neovascularization and inflammation and support the rationale for targeting their structure–function relationships in the development of novel antiangiogenic therapies.

The advent of anti-VEGF therapies has represented a major breakthrough in the management of neovascular age-related macular degeneration (AMD), diabetic retinopathy, and retinal vein occlusion by targeting vascular endothelial growth factor (VEGF), one of the most potent endogenous inducers of angiogenesis [15,16,17]. These therapies have led to significant improvements in visual outcomes [18]. However, their long-term efficacy tends to plateau in many patients, and the need for frequent intravitreal injections imposes a substantial treatment burden, negatively impacting patient adherence and quality of life [19]. In addition, anti-VEGF agents are expensive and inaccessible to the underserved populations residing mainly in developing countries, limiting their global impact. Moreover, the systemic administration of anti-VEGF agents—particularly in oncology—has been associated with serious adverse effects, including hypertension and renal dysfunction, and emerging evidence suggests that systemic effects may also arise with chronic local administration [20]. Given the critical role of the VEGF system in vascular homeostasis and the clinical outcome of anti-VEGF therapies, current strategies remain a cornerstone in the treatment of neovascular eye diseases. Nevertheless, the limitations related to this treatment have stimulated growing interest in identifying alternative or complementary target pathways involved in pathological angiogenesis.

Heparin and its structural mimetics have emerged as promising agents for the modulation of pathological angiogenesis through mechanisms that are not limited to direct VEGF inhibition. These compounds influence several signaling pathways involved in endothelial proliferation, migration, and neovessel formation, thus acting at multiple levels of the angiogenic cascade [21,22]. Although the clinical use of native heparin is limited by its strong anticoagulant and hemorrhagic potential, chemically modified derivatives and mimetics have been shown to retain antiangiogenic activity while minimizing coagulation-related risks [22]. Recent studies have demonstrated that such compounds can inhibit choroidal neovascularization and modulate fibroblast growth factor (FGF) and VEGF signaling in ocular models, supporting their use as complementary or alternative strategies to current anti-VEGF therapies [21,22]. These findings highlight a valuable therapeutic avenue for improving efficacy, safety, and accessibility in the treatment of age-related macular degeneration and other vascular proliferative retinopathies.

This study aims to explore the therapeutic potential of 6-O-desulfated heparin, designed to retain antiangiogenic activity with reduced anticoagulant effects. Heparin was selected due to its well-documented ability to modulate angiogenesis and inflammation through specific interactions with key molecular mediators. We assessed the cytocompatibility of 6-O-desfulated heparin (Hep-6Od) in retinal pigment epithelial cells via in vitro cytotoxicity assays. Moreover, the effects of Hep-6Od on endothelial cell proliferation, migration, and capillary tube formation were then evaluated to characterize its anti-angiogenic profile. Additionally, biochemical interaction assays were conducted to examine its binding with FGF-2, a central mediator of neovascularization. Finally, its efficacy was tested in vivo using an animal model of laser-induced choroidal neovascularization, where Hep-6Od was administered via intravitreal injection. By targeting complementary pathways in angiogenesis, this study aims to contribute to the development of better, safer and more accessible therapeutic options for retinal vascular diseases such as neovascular AMD.

## 2. Results

### 2.1. Structural Confirmation of 6-O-Desulfated Heparin by NMR (^13^C/^1^H)

**Figure 1:**
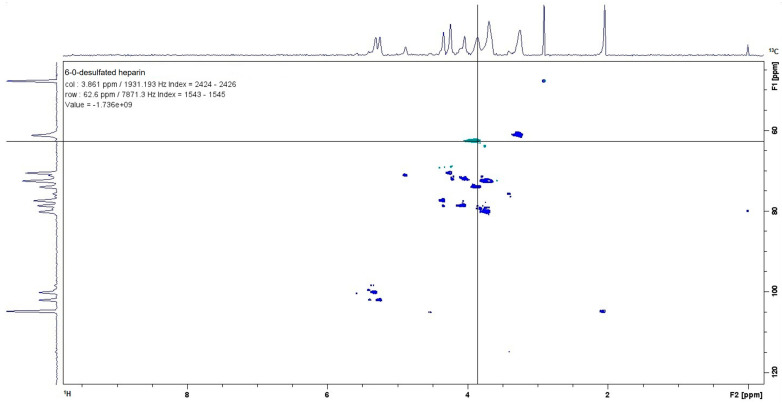
The modified heparin structure was confirmed using a nuclear magnetic resonance (NMR) analysis. The HSQC spectrum provided a direct correlation between the ^13^C signals (*Y*-axis) and the ^1^H signals (*X*-axis). This observation verifies that the desulfation at the 6-O position was successfully achieved, thereby validating the structure of the 6-O-desulfated heparin.

After the selective chemical modifications of porcine heparin to prepare it for subsequent experiments, the chemical structure of 6-O-desulfated heparin was confirmed using Nuclear Magnetic Resonance (HSQC—Heteronuclear Single Quantum Coherence) with a Bruker Ascend 500 MHz spectrometer (Bruker Corporation, Billerica, MA, USA). This experiment analyzed both ^13^C and ^1^H heteronuclear spectra. Specifically, according to Yates et al. [23], the ^13^C signal at 62.6 parts per million (ppm) corresponded to carbon 6 (C6) of glucosamine, while the ^1^H signal at 3.861 ppm indicated that this carbon was bonded to a hydrogen atom. As a result, the experiment successfully demonstrated that desulfation at the C6 position of glucosamine was achieved, confirming the structure of 6-O-desulfated heparin (Figure 1).

### 2.2. Coagulation Assays

**Figure 2:**
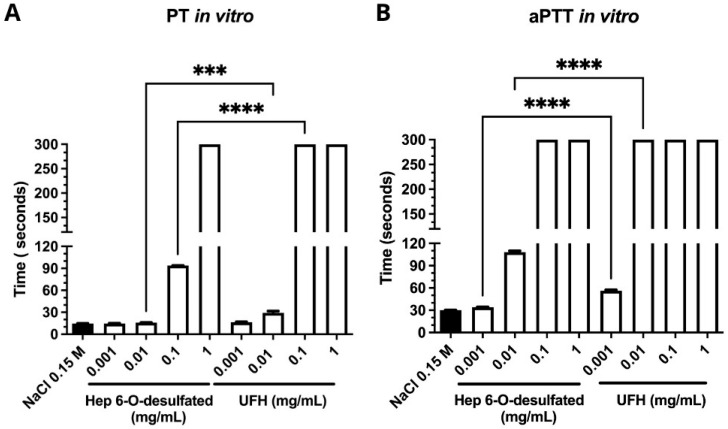
In vitro anticoagulant and antihemorrhagic activities of 6-O-desulfated heparin (Hep-6Od) and unfractionated heparin (UFH) performed in biological triplicates (*n* = 3) and analyzed using unpaired two-tailed *t*-tests. (**A**) Prothrombin time (PT) assay evaluating the extrinsic coagulation pathway. The results show that PT values for 6-O-desulfated heparin at various concentrations were lower than the corresponding concentrations of UFH. (**B**) Activated partial thromboplastin time (aPTT) assay assessing the intrinsic coagulation pathway. The findings demonstrate that 6-O-desulfated heparin exhibited shorter aPTT values at equivalent concentrations compared to UFH. The magnitude of statistical significance is indicated by asterisks for comparisons between equivalent doses of Hep-6Od and UFH. *** corresponds to *p* < 0.001; **** indicates *p* < 0.0001.

The prothrombin time (PT) assay, targeting the extrinsic pathway of blood coagulation, revealed that Hep-6Od concentrations of 0.001 mg/mL and 0.01 mg/mL exhibited average clot formation times of 14.6 s and 15.9 s, respectively. In comparison, unfractionated heparin (UFH) showed clot formation times of 16.4 s and 29.1 s at the same concentrations. At concentrations of 0.1 mg/mL and 1 mg/mL, Hep-6Od displayed clot formation times of 93.8 s and 300 s, respectively, whereas UFH samples exhibited a clot formation time of 300 s (Figure 2A). The activated partial thromboplastin time (aPTT) assay, which evaluates the intrinsic pathway of blood coagulation (involving factors VIII, IX, XI, and XII) as well as common pathway factors (fibrinogen, prothrombin, and factors V and X), demonstrated that 6-O-desulfated heparin at concentrations of 0.001 mg/mL and 0.01 mg/mL resulted in average clot formation times of approximately 33.9 s and 34.8 s, respectively. At a concentration of 0.1 mg/mL, the clot formation time increased to 108.2 s, while at 1 mg/mL, the clotting time exceeded 300 s.

In contrast, the lowest concentration of unfractionated heparin (UFH) tested (0.001 mg/mL) resulted in an average clot formation time of 56.3 s, whereas all higher concentrations (≥0.01 mg/mL) produced clotting times greater than 300 s (Figure 2B).

### 2.3. Viability of Rabbit Aortic Endothelial Cells (RAEC) and Retinal Pigment Epithelial Cells (ARPE-19)

The viability of rabbit aortic endothelial cells (RAEC) and retinal pigment epithelial cells (ARPE-19) was assessed using the MTT assay. Cells were treated with 6-O-desulfated heparin at concentrations of 0.001 mg/mL, 0.01 mg/mL, and 0.1 mg/mL, while control groups received phosphate-buffered saline (PBS) at the same volume as Hep-6Od.

**Figure 3:**
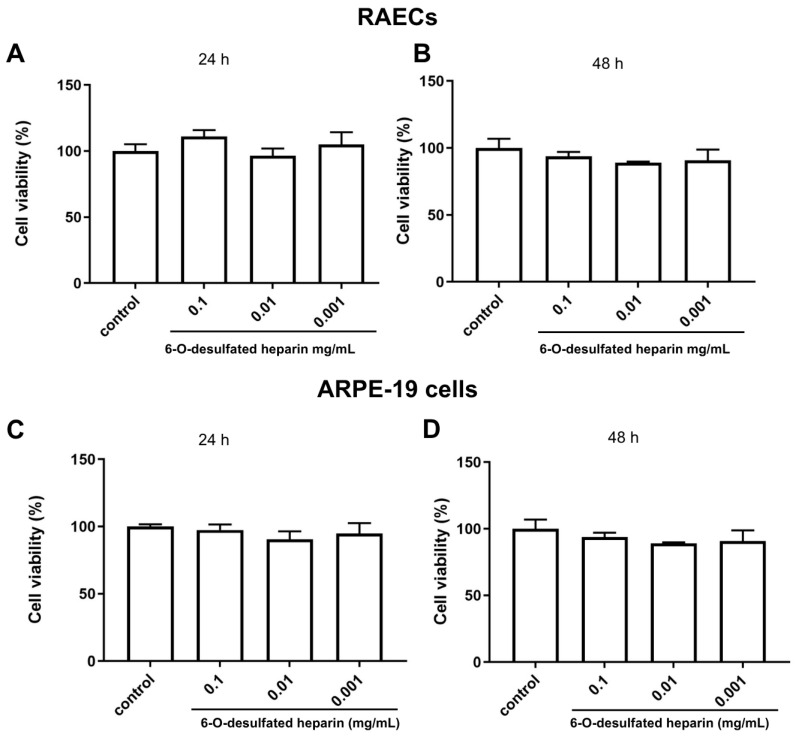
In vitro analysis of cell viability after treatment with 6-O-desulfated heparin in rabbit aortic endothelial cells (RAECs) and retinal pigment epithelial cells (ARPE-19 cells). Cell viability was assessed using the MTT colorimetric assay. (**A**) In RAECs, after 24 h of treatment, no concentration of 6-O-desulfated heparin significantly affected cell viability compared to the controls (*p* = 0.0979). (**B**) After 48 h, RAEC viability also remained unaffected by the treatment (*p* = 0.1564). (**C**) In ARPE-19 cells, no significant differences in cell viability were observed at 24 h following treatment (*p* = 0.0747). (**D**) Similarly, after 48 h, ARPE-19 cell viability was not significantly impacted by any concentration of 6-O-desulfated heparin compared to the controls (*p* = 0.9671). The statistical analysis was performed using one-way ANOVA followed by post hoc testing.

The statistical analysis was performed using one-way ANOVA followed by post hoc testing to compare all 6-O-desulfated heparin-treated groups to the saline control group. In RAECs, no significant differences in viability were observed at either 24 or 48 h, with *p*-values of 0.0979 and 0.1564, respectively (Figure 3A,B).

Similarly, for ARPE-19 cells, treatment with 6-O-desulfated heparin did not significantly alter viability compared to controls at 24 or 48 h, with *p*-values of 0.0747 and 0.9671, respectively (Figure 3C,D).

These results suggest that 6-O-desulfated heparin does not significantly impact the viability of RAEC or ARPE-19 cells under the tested conditions.

### 2.4. Cellular Proliferation

Cellular proliferation was assessed by the direct counting of nuclei stained using DAPI (4′,6-diamidino-2-phenylindole), allowing the quantification of the total number of cells. Treatments with 6-O-desulfated heparin (0.001, 0.01, and 0.1 mg/mL) and a control treatment with phosphate-buffered saline (PBS) were performed for 24 and 48 h.

**Figure 4:**
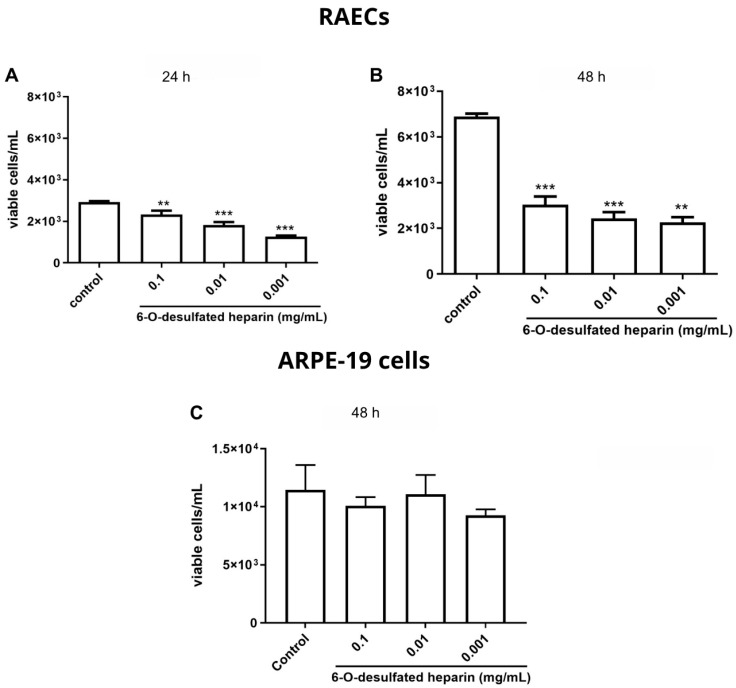
Analysis of the proliferation of rabbit aortic endothelial cells (RAECs) and retinal pigmented epithelial cells (ARPE-19 cells). (**A**) In RAECs, after 24 h of treatment, concentrations of 0.1 mg/mL, 0.01 mg/mL, and 0.001 mg/mL of 6-O-desulfated heparin resulted in decreases in cell counts by 22.58%, 37.7%, and 43.1%, respectively, compared to the control. (**B**) After 48 h, reductions in RAEC counts relative to the control were 53.62%, 65%, and 68% for the corresponding concentrations. (**C**) At 48 h, ARPE-19 cell proliferation showed no significant differences compared to the control. The *p*-value was recorded at 0.1849. Statistical significance was assessed using one-way ANOVA followed by the Bonferroni post hoc analysis, with significance levels indicated as ** *p* < 0.01 and *** *p* < 0.0001.

The impact of 6-O-desulfated heparin on cellular proliferation was evaluated in RAECs and the ARPE-19 cell line (Figure 4). In RAECs, all tested concentrations of 6-O-desulfated heparin significantly reduced cell proliferation compared to the control group (PBS) at both 24 and 48 h of treatment, with *p* values < 0.05. At 24 h, treatment with 0.1 mg/mL resulted in a 22.58% reduction in the number of cells, 0.01 mg/mL led to a 37.7% decrease, and 0.001 mg/mL caused a 43.1% reduction. At 48 h, the reductions observed were 53.62% for 0.1 mg/mL, 65% for 0.01 mg/mL, and 68% for 0.001 mg/mL. These findings indicate that lower concentrations of 6-O-desulfated heparin were more effective at inhibiting RAEC cell proliferation over time (Figure 4A,B).

On the other hand, Hep-6Od did not significantly influence ARPE-19 cell proliferation at any of the tested concentrations (0.1 mg/mL, 0.01 mg/mL, and 0.001 mg/mL) compared to the control group (PBS) at both 24 and 48 h. The observed *p*-value of 0.1849 indicates that the changes in the total number of cells were not statistically significant under the experimental conditions (Figure 4C).

### 2.5. Endothelial Tube Formation Assay After the Treatment of RAECs with Hep-6Od

The endothelial tube formation assay demonstrated that the treatment of RAECs with different concentrations of 6-O-desulfated heparin (Hep-6Od) significantly reduced the formation of capillary-like structures in culture, with the results compared to the control (PBS). At a concentration of 0.01 mg/mL, 6-O-desulfated heparin led to an approximately 84% reduction in total tube length compared to the control (*p* < 0.05). Similarly, at 0.001 mg/mL, a substantial reduction of approximately 72% was observed (*p* < 0.05). However, despite showing a small reduction in capillary-like structure formation, the highest concentration tested, 0.1 mg/mL, did not yield a statistically significant decrease in total tube length.

**Figure 5:**
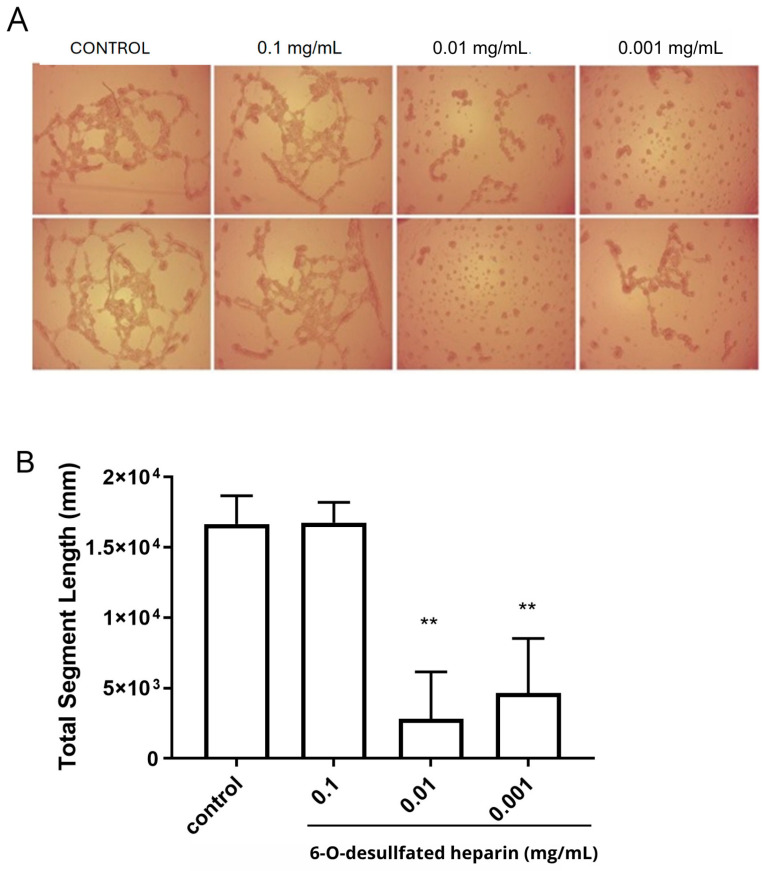
In vitro endothelial tube formation by rabbit aortic endothelial cells (RAECs) cultured on Matrigel and treated with different concentrations of Hep-6Od or PBS (control). (**A**) After 16 h of treatment with Hep-6Od, RAECs showed a significant decrease in capillary tube formation at all tested concentrations. Images were captured at 40× magnification using an inverted light microscope (Zeiss Primovert, Carl Zeiss Microscopy, Jena, Germany). (**B**) The findings revealed a marked decrease in the total length of endothelial tube segments at heparin concentrations of 0.01 mg/mL (84% reduction) and 0.001 mg/mL (72% reduction) compared to the control, with both reductions being statistically significant (*p* < 0.05). In contrast, the 0.1 mg/mL concentration did not induce a statistically significant reduction in tube formation. ** *p*-values were determined using one-way ANOVA and the Bonferroni correction.

When comparing the concentrations of 0.01 mg/mL and 0.001 mg/mL, no statistically significant difference was observed in their effects on endothelial tube formation (Figure 5A,B).

These findings underline the concentration-dependent effects of Hep-6Od on endothelial tube formation, suggesting its potential antiangiogenic properties. Notably, lower concentrations of Hep-6Od were more effective at suppressing endothelial tube formation than the higher dose.

### 2.6. Wound Healing Assay

**Figure 6:**
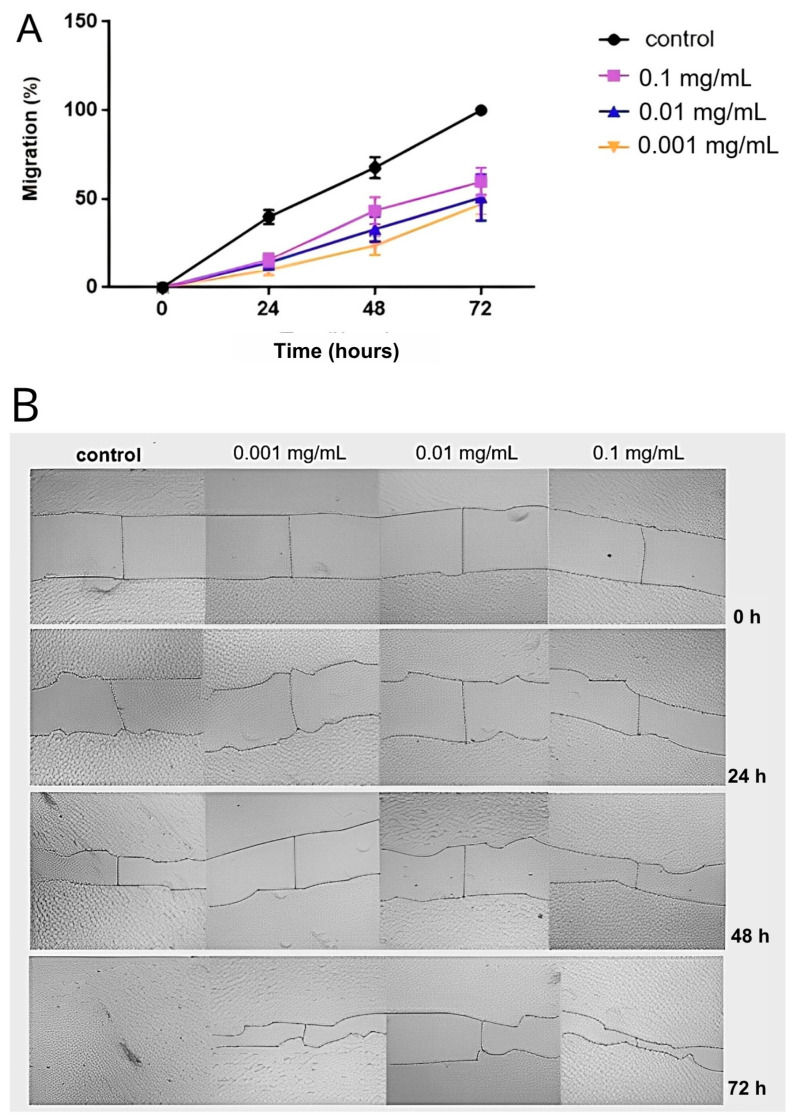
Wound healing assay of rabbit aortic endothelial cells (RAEC) treated with Hep-6Od. (**A**) The migration rate of endothelial cells in response to injury was quantitatively assessed at various intervals (0 h, 24 h, 48 h, and 72 h), with 0% migration at 0 h and 100% migration at 72 h as benchmarks of the control group (PBS). The results revealed that all three tested concentrations of Hep-6Od (0.1 mg/mL, 0.01 mg/mL, and 0.001 mg/mL) significantly influenced cellular migration compared to the control, with *p*-values < 0.0001, as determined by one-way ANOVA followed by Bonferroni correction. (**B**) Bright-field images of RAECs were captured at 0 h, 24 h, 48 h, and 72 h following treatment with three different concentrations of 6-O-desulfated heparin or PBS (control). These observations were made using a bright-field inverted microscope (Zeiss Primovert, Carl Zeiss Microscopy, LLC., Jena, Germany) at 10× magnification.

The cell migration assay performed by a wound healing assay of RAECs after treatment with different concentrations of Hep-6Od or PBS (control) demonstrated a significant inhibitory effect of 6-O-desulfated heparin on cellular migration, highlighting its potential role in modulating vascular responses to injurious stimuli. At a concentration of 0.1 mg/mL, Hep-6Od notably reduced cellular migration, with migration rates of 15.51% at 24 h, 43.4% at 48 h, and 59.6% at 72 h. Similarly, at 0.01 mg/mL, migration rates were reduced to 14.02% at 24 h, 32.9% at 48 h, and 50.78% at 72 h. The lowest concentration tested, 0.001 mg/mL, resulted in migration rates of 9.8% at 24 h, 23.6% at 48 h, and approximately 47% at 72 h, all of which were significantly lower than the control (*p* < 0.001) that showed 43%, 68%, and 100% closure at 24 h, 48 h, and 72 h (Figure 6). These results highlight the dose-dependent inhibitory effects of Hep-6Od on cellular migration.

### 2.7. Molecular Interaction Assay of 6-O-Desulfated Heparin and FGF-2 by DSF

**Figure 7:**
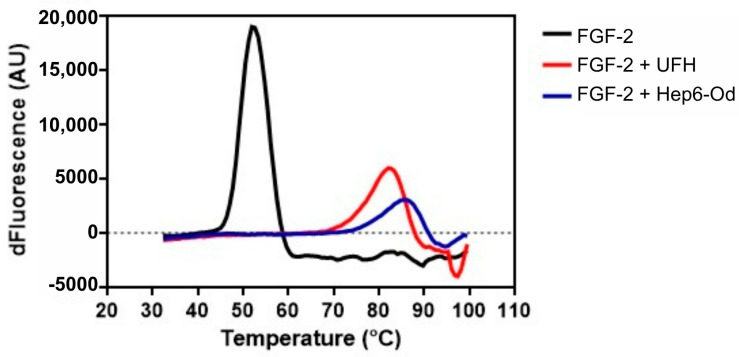
Effect of the thermal stabilization of FGF2 in the presence of unfractionated heparin and 6-O-desulfated heparin. The thermal stability of FGF2 (0.2 mg/mL) was evaluated using differential scanning fluorimetry (DSF) in the presence of unfractionated heparin (10 mg/mL) and 6-O-desulfated heparin (10 mg/mL).

Differential scanning fluorometry (DSF) was employed to evaluate the thermal stability of FGF2 by monitoring protein unfolding through fluorescence changes using the Sypro^®^ dye. The melting temperature (Tm) for FGF2 alone was determined to be 51.95 °C, reflecting its folded state. Upon complexation with heparin (UFH), the Tm increased significantly to 82 °C, indicating enhanced stabilization via the exposure of hydrophobic regions. Notably, the interaction of FGF2 with 6-O-desulfated heparin further raised the Tm to 85.5 °C, suggesting that C6-desulfation of glucosamine marginally improved the thermal stability of FGF2 compared to unmodified heparin. These findings underscore the role of heparin interactions in modulating FGF2 stability (see Table 1 and Figure 7).

### 2.8. Hep6-Od Treatment of Laser-Induced Choroidal Neovascularization, an In Vivo Assay

To evaluate the in vivo efficacy of 6-O-desulfated heparin (Hep-6Od) in modulating choroidal neovascularization (CNV), a laser-induced CNV model was employed. Following the induction of CNV by laser photocoagulation, 6-O-desulfated heparin was intravitreally injected at three different doses: 0.05 μg, 0.5 μg, and 5 μg. The neovascular areas were subsequently assessed by immunofluorescence staining, allowing for the precise visualization of the neovascularized area.

**Figure 8:**
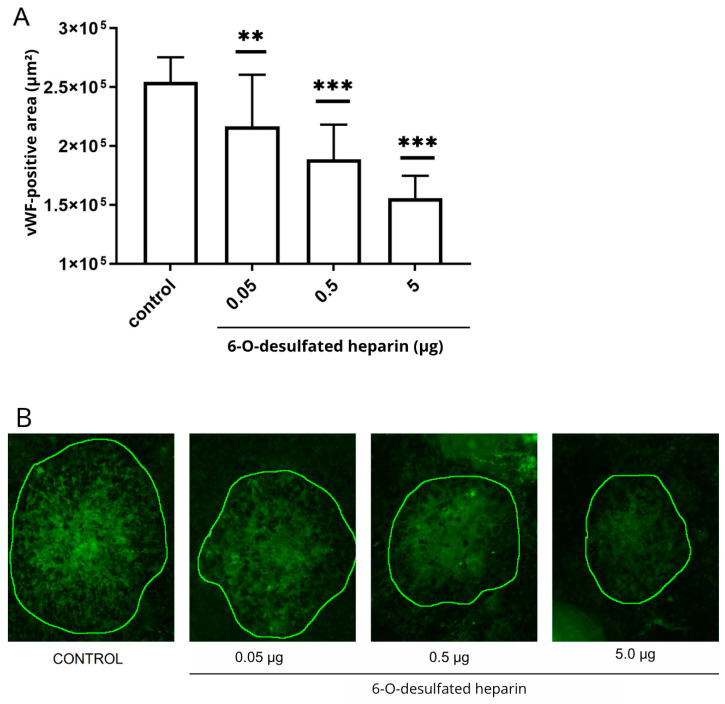
Choroidal neovascularization (CNV) area following the intravitreal injection of Hep-6Od in a laser-induced CNV model in Lean Zucker rats. (**A**) The intravitreal injection of 6-O-desulfated heparin resulted in reductions in the neovascularized area of 19.23%, 26.92%, and 44.23% for the doses of 0.05 µg, 0.5 µg, and 5 µg, respectively, compared to the control group. Statistically significant differences were observed (** *p* < 0.01 and *** *p* < 0.001, one-way ANOVA followed by the Bonferroni post hoc test). (**B**) Flat-mounted choroidal specimens were immunostained for von Willebrand factor (vWF) to delineate neovascularized areas and examined under an inverted fluorescence microscope (Axio Observer Z1 microscope—Zeiss, Jena, Germany) at 10× magnification. The scale bar represents 100 µm.

The results revealed a significant reduction in the neovascular lesion area after treatment with all tested concentrations when compared to the control (EBSS) (*p* < 0.001). Specifically, the 0.05 μg dose resulted in a 14.7% reduction (*p* < 0.05), the 0.5 μg dose achieved a 25.7% reduction (*p* < 0.01), and the 5 μg dose exhibited the most substantial effect, with a 38.7% decrease in neovascularization (*p* < 0.001) (Figure 8). These findings suggest that 6-O-desulfated heparin effectively attenuates CNV, with the 5 μg dose demonstrating the highest efficacy in reducing neovascularized area.

## 3. Discussion

Age-related macular degeneration (AMD) remains a leading cause of irreversible vision loss among older adults in developed countries, with choroidal neovascularization (CNV) constituting the principal pathological hallmark of the neovascular (wet) form of the disease [2]. Although anti-vascular endothelial growth factor (anti-VEGF) therapies have improved visual outcomes and are currently the standard of care, significant limitations persist, including the need for repeated intravitreal injections, the potential development of tachyphylaxis, partial or non-responsiveness in a subset of patients, and the associated treatment burden [18,19]. These challenges highlight the critical need for adjunct or alternative therapeutic strategies that can enhance or replace the current regimens.

Heparin, a highly sulfated glycosaminoglycan, is traditionally recognized for its potent anticoagulant and anti-inflammatory activities [9,10]. Recent research has expanded interest in heparin’s role as a modulator of angiogenesis, particularly in ocular neovascular disorders. However, the systemic use of native heparin is constrained by a significant risk of hemorrhagic complications. To circumvent these limitations, chemically modified derivatives of heparin have been developed, aiming to retain or enhance the antiangiogenic efficacy while minimizing anticoagulant activity [21].

In this context, 6-O-desulfated heparin (Hep-6Od), a selectively modified form of heparin, emerges as a promising candidate. Prior studies have demonstrated that selective desulfation can significantly attenuate anticoagulant properties without abolishing the antiangiogenic potential. Based on this rationale, we investigated the therapeutic efficacy of 6-O-desulfated heparin in a preclinical model of CNV, seeking to explore its ability to suppress pathological neovascularization—a central feature in the pathogenesis of neovascular AMD.

In vitro assays provided robust evidence supporting the antiangiogenic potential of 6-O-desulfated heparin (Hep-6Od). The compound significantly inhibited key cellular processes involved in angiogenesis, including endothelial cell proliferation and migration. DAPI-based nuclear counts revealed a marked reduction in cell proliferation following treatment with Hep-6Od at all tested concentrations, while wound healing assays demonstrated a potent and dose-dependent inhibition of RAEC migration over 72 h. Importantly, MTT viability assay indicated no cytotoxicity, similar result of retinal cells. Together, these results suggest that Hep-6Od effectively impairs essential angiogenic mechanisms. Moreover, coagulation assays (PT and aPTT) demonstrated that Hep-6Od exhibited significantly reduced anticoagulant activity compared to unfractionated heparin (UFH), further supporting its safety profile for potential therapeutic applications targeting pathological angiogenesis, such as in neovascular eye diseases.

For instance, Kniggendorf et al. [22] demonstrated that a chemically modified heparin, N-desulfated re-N-acetylated heparin (mHep), exerted antiproliferative and antimigratory effects on endothelial cells in vitro. Similarly, Dreyfuss et al. [21] reported that a heparin mimetic isolated from marine shrimp suppressed neovascularization in vitro and in vivo.

Interestingly, the dose–response behavior of Hep-6Od in the in vitro assays did not follow a linear pattern. In both cell proliferation and tube formation assays, the lower concentration (10 µg/mL) appeared to exert a more potent antiangiogenic effect than higher doses. This phenomenon is consistent with the concept that angiogenesis is regulated by highly dynamic and concentration-sensitive interactions between glycosaminoglycans and angiogenic mediators. Heparins modulate this process by influencing the spatial availability and receptor engagement of key growth factors such as FGF2 and VEGF, as well as by regulating inflammatory cytokines and matrix-bound signaling reservoirs. At lower doses, Hep-6Od may more effectively sequester growth factors or prevent their binding to receptors, whereas at higher doses, saturation effects, altered electrostatic interactions, or compensatory cellular responses may diminish efficacy.

This nonlinear response has been previously observed in our group’s work with other chemically modified heparins, where lower doses frequently produced more pronounced biological effects. Moreover, similar patterns have been reported in the literature. Dreyfuss et al. [21] described a marine-derived heparin mimetic with reduced anticoagulant activity that more effectively inhibited neovascularization at intermediate concentrations. Likewise, Kniegendorf et al. [22] demonstrated that structural changes in modified heparins significantly influence their antiangiogenic activity in a dose-dependent yet nonlinear manner. Together, these findings reinforce the importance of fine structural tuning and concentration optimization in the development of heparin-based antiangiogenic therapies.

Beyond the cellular assays, our protein thermal stability studies by DSF provided critical insights into molecular interactions. Specifically, we demonstrated a direct binding between Hep-6Od and FGF-2, a key proangiogenic growth factor. This interaction appears to interfere with the FGF-2 signaling cascade, a pathway intricately involved in neovascularization, and is consistent with the established concept that native heparin and its derivatives can modulate the bioavailability and receptor binding of various growth factors [9]. These findings suggest that the antiangiogenic effects of Hep-6Od are mediated, at least in part, through the disruption of growth factor signaling networks.

Heparan sulfate proteoglycans (HSPGs) are key regulators of cellular signaling, broadly influencing cell proliferation, migration, adhesion, and apoptosis through their interactions with a wide array of proteins, including growth factors, morphogens, extracellular matrix components, cytokines, and enzymes. Their structurally diverse GAG chains act as critical modulators of the extracellular environment, facilitating or restraining the signaling events essential for tissue homeostasis and pathological processes like angiogenesis [10].

In the context of angiogenesis, heparan sulfate chains function predominantly as co-receptors, presenting, facilitating and stabilizing the binding of growth factors such as VEGF, FGF-2, HGF, and PDGF to their high-affinity receptors. In addition Heparan sulfate provides a reservoir of readily available growth factors within the ECM. This organization facilitates receptor dimerization and activation, triggering downstream pathways such as MAPK, PI3K/Akt, and PLCγ, which collectively drive endothelial cell proliferation, migration, survival, and cytoskeletal reorganization. The sulfation patterns of heparan sulfate, particularly at the N-, 2-O-, and 6-O- positions, are critical for these interactions, dictating the selectivity and strength of protein binding [24,25].

The chemically modified heparin derivative Hep-6Od, characterized by selective 6-O-desulfation, acts as a heparan sulfate mimetic that may strategically disrupt these protein–GAG interactions without abolishing the overall binding affinity. By maintaining the ability to bind growth factors like FGF-2 but impairing proper signaling complex formation, Hep-6Od effectively attenuates endothelial cell responses essential for angiogenesis, as shown in this work. This disruption inhibits endothelial cell proliferation, migration, and capillary-like tube formation, as demonstrated in both in vitro assays and in vivo models of choroidal neovascularization.

Moreover, heparan sulfate not only serves as a platform for growth factor signaling but also regulates endocytic uptake pathways and the spatial distribution of morphogens, chemokines, and cytokines [25]. Alterations in the heparan sulfate structure, such as those introduced by selective desulfation, can modulate these pathways, impacting the cellular dynamics crucial for angiogenic progression [25,26]. By mimicking an under-sulfated heparan sulfate environment, Hep-6Od decreases growth factor bioavailability and signal transduction, thus shifting the cellular balance toward decreased proliferation, impaired migration, and potentially enhanced apoptosis.

In vivo, a dose-dependent reduction in CNV lesion areas was observed following the intravitreal injection of Hep-6Od in a laser-induced CNV model in lean Zucker rats. The concentrations of Hep-6Od used in vitro were selected to reflect the estimated intraocular levels achieved after an intravitreal injection in Zucker rats. Based on a vitreous volume of 50 μL [27,28], we calculated that doses of 0.5 to 5.0 μg/eye correspond to final concentrations of approximately 10 to 100 μg/mL, which were matched in the in vitro assays to support translational relevance.

Although UFH was used as a reference compound in the anticoagulant assays, it was not included in the in vivo experiments. This decision was based on prior observations in our laboratory showing that an intravitreal injection of UFH in Zucker rats induces severe intraocular hemorrhage, making it incompatible with subsequent imaging, lesion quantification, and animal welfare standards. Given its high anticoagulant potency, UFH proved to be excessively hemorrhagic when administered intraocularly. For these reasons, and in accordance with the ethical principles of animal experimentation, we opted not to include UFH in the in vivo arm of this study. The reduced hemorrhagic potential of Hep-6Od reinforces its safety profile and translational relevance for ocular applications.

This finding provides further evidence for the antiangiogenic activity of Hep-6Od and suggests that it may be effective at suppressing neovascularization in vivo. The reduction in the CNV area observed in our study is consistent with the results reported by Dreyfuss et al. [21] and Kniggendorf et al. [22], who found that a heparin mimetic isolated from marine shrimp and a chemical modification of UFH heparin, respectively, reduced the CNV area in a similar animal model.

Collectively, these findings validate the antiangiogenic activity of Hep-6Od, rooted not merely in its capacity to interfere with specific sulfation-dependent growth factor interactions but in a broader capacity to modulate the heparan sulfate-dependent regulation of cellular behavior. This highlights Hep-6Od as a promising therapeutic agent to target pathological neovascularization through mechanisms extending beyond VEGF inhibition.

This study provides compelling evidence for the antiangiogenic potential of Hep-6Od, and may have important clinical implications in the future studies. The fact that Hep-6Od exhibited antiangiogenic activity without significant cytotoxicity to ARPE-19 cells suggests that it may be a safe therapy for the treatment of wet AMD. Furthermore, the ability of 6-OHep to interact with FGF-2 suggests that it may be effective in patients who are non-responsive to anti-VEGF therapies, as it targets a different signaling pathway involved in angiogenesis.

Future studies should focus on validating the efficacy and safety of 6-OHep in other animal models and, ultimately, in human clinical trials. In addition, further research is needed to elucidate the precise mechanisms by which Hep-6Od exerts its antiangiogenic effects, including its interactions with other growth factors and signaling pathways.

In conclusion, our study provides compelling evidence for the antiangiogenic potential of 6-OHep and suggests that it may be a promising new therapeutic agent for the treatment of wet AMD. Further research is warranted to fully explore its clinical potential and to optimize its use in combination with existing therapies.

## 4. Materials and Methods

### 4.1. Synthesis and Characterization of Modified Heparin

**Figure 9:**
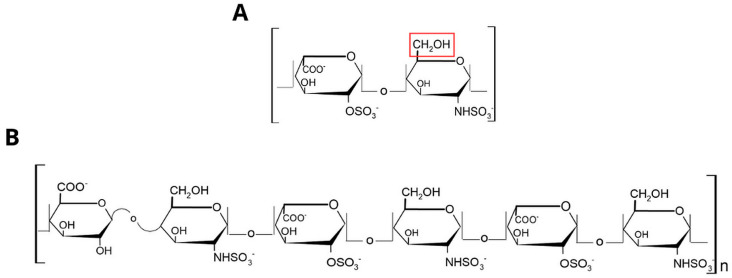
Chemical structure of the 6-O-desulfated heparin. (**A**) The major disaccharide unit of heparin, which corresponds typically to 70–80%. The disaccharide consists of an α-L-iduronic acid linked by an α(1,4) bond to a 6-O-desulfated glucosamine. The red square highlights the carbon in glucosamine where desulfation was performed (adapted from Meneghetti, et al. [29]). (**B**) The hexasaccharide chain of 6-O-desulfated heparin is described, with the initial disaccharides formed by a non-sulfated β-D-glucuronic acid (GlcA) linked to an N-sulfated glucosamine (GlcNSO_3_) via a β 1,4 bond, and subsequent disaccharides consisting of a 2-O-sulfated α-L-iduronic acid (IdoA2SO_3_) and an N-sulfated glucosamine (GlcNSO_3_).

Unfractionated heparin (UFH) from porcine intestinal mucosa (Extrasul, Jaguapita, Brazil) was used to generate the 6-O-desulfated heparin according to the procedures outlined in the protocol developed by Yates et al. in 1996 [23] (Figure 9).

Initially, a Dowex 50WX8 ion-exchange column was prepared with 1 M hydrochloric acid, then washed to remove free protons. UFH was passed through the column, washed again, eluted and its pH adjusted to 7.0 using pyridine. The resulting pyridine salt-form heparin solution was frozen and lyophilized for storage.

Selective O-desulfation at the C6 position and N-desulfation at the C2 position of glucosamine residues were performed by solvolytic desulfation of the heparin–pyridine salt in a DMSO/methanol (9:1) mixture at 60 °C for 4 h. The pH was adjusted to 7.0 with NaOH. The reaction product was precipitated with cold ethanol saturated with sodium acetate at 4 °C for 16 h. Selective O-desulfation occurs predominantly at the C6 position of the glucosamine units, along with partial N-desulfation at the C2 position, due to the lability of the N-sulfate group. Consequently, the next step involved N-resulfation in this position. N-resulfation at C2 of glucosamine was carried out by reacting the partially desulfated heparin with a trimethylamine–sulfur trioxide complex in the presence of sodium bicarbonate, adjusting the pH to 9.0. Following the reaction, the modified heparin was precipitated again with cold ethanol saturated with sodium acetate and incubated at 4 °C for 16 h. Afterwards the precipitate was desalted by gel filtration chromatography using an FPLC system (GE Healthcare, Uppsala, Sweden).

### 4.2. Characterization of 6-O-Desulfated Heparin by ^13^C–^1^H NMR

The chemically modified 6-O-desulfated heparin was dissolved in deuterium oxide (D_2_O, 99.9% atom D; Cambridge Isotope Laboratories Inc., Andover, MA, USA) at a final concentration of 10 mg/mL. The NMR spectra were acquired at 22 °C using a Bruker Ascend 500 MHz spectrometer (Billerica, MA, USA) [30].

### 4.3. Coagulation Assay

#### 4.3.1. Prothrombin Time (PT) Determination

The prothrombin time assessment utilized a semi-automatic coagulometer (BFT II—Dade Behring) with reconstituted lyophilized rabbit brain thromboplastin from Dade Behring. The procedure involved combining 50 μL of human pooled plasma with 50 μL of either a saline solution (as control) or various concentrations of 6-O-desulfated heparin (6-OHep) (ranging from 1 mg/mL to 0.001 mg/mL) in tubes maintained at 37 °C. Experiments were performed in biological triplicates (*n* = 3) and analyzed using unpaired two-tailed *t*-tests. Following a 60 s incubation, 100 μL of thromboplastin was added. The experiments, conducted in duplicate, measured clotting times up to 300 s, providing quantitative data on heparin’s anticoagulant properties.

#### 4.3.2. Activated Partial Thromboplastin Time (aPTT) Determination

The activated partial thromboplastin time (aPTT) was determined using a semi-automatic coagulometer (BFT II—Dade Behring). Activated cephalin derived from rabbit brain, containing celite as the activator, was used by following the procedure described by Silva et al. [31]. In a standardized tube preheated to 37 °C, 50 µL of plasma and 50 µL of cephalin (with either 50 µL of saline as a control or one of various concentrations of 6-O-desulfated heparin [1 mg/mL, 0.1 mg/mL, 0.01 mg/mL, or 0.001 mg/mL]) were added. Experiments were performed in biological triplicates (*n* = 3) and analyzed using unpaired two-tailed *t*-tests After an incubation at 37 °C for 120 s, 50 µL of 0.025 M CaCl_2_, preheated to 37 °C, was added while simultaneously starting the timer. Measurements were performed in duplicate, and the results were expressed as the clotting time for the different plasma tests. Clotting times are reported in seconds, with the maximum assay duration set at 300 s.

### 4.4. Cell Culture

In vitro experiments employed two cell lineages: rabbit aortic endothelial cells (RAECs) and retinal pigment epithelial cells (ARPE-19 cells). These cell lines were cryogenically preserved in 1 mL tubes containing culture medium supplemented with 20% fetal bovine serum (FBS) (Cultilab, Campinas, São Paulo, Brazil) and 10% dimethyl sulfoxide (DMSO) (*v*/*v*) from Aldrich Chemical Company, Inc. (Milwaukee, WI, USA) in liquid nitrogen.

Rabbit aortic endothelial cells (RAECs) were cultured in F-12 culture medium (Gibco^®^, Life Technologies, Rockville, MD, USA) enriched with 10% fetal bovine serum (FBS), 20 mM sodium bicarbonate, and 1% penicillin/streptomycin (Sigma Chemical Co., St. Louis, MO, USA) in adhesive culture dishes (100 mm × 20 mm) (BD Falcon^TM^, San Jose, CA, USA). They were incubated at 37 °C in a 2.5% CO_2_ atmosphere [9].

Adult retinal pigment epithelial cells (ARPE-19 cells) were maintained in DMEM/F12 medium (Life Technologies/Gibco^®^, Rockville, MD, USA) supplemented with 10% fetal bovine serum (FBS; Cultilab, Campinas, SP, Brazil), 15 mM HEPES, 2.0 mM L-glutamine, 0.5 mM sodium pyruvate, 20 mM sodium bicarbonate, and 1% penicillin/streptomycin (Sigma Chemical Co., St. Louis, MO, USA). Cultures were plated in culture dishes (BD Falcon^TM^, San Jose, CA, USA) and incubated at 37 °C in a humidified atmosphere containing 5.0% CO_2_, ensuring a proper pH balance.

### 4.5. Cell Viability (Cytotoxicity) Assay

To assess cytotoxicity, the MTT (3-(4,5-dimethylthiazol-2-yl)-2,5-diphenyltetrazolium bromide) assay was employed. RAECs and ARPE-19 cells were initially seeded in 96-well plates at densities of 2.5 × 10^4^ and 5.0 × 10^4^ cells per well, respectively. After five days, the cultures were treated with 6-O-desulfated heparin at concentrations of 0.001 mg/dL, 0.01 mg/dL, or 0.1 mg/dL prepared in a culture medium supplemented with 10% FBS. After the treatment periods, the culture medium was removed, and the wells were washed with a phosphate-buffered saline (PBS) solution. Subsequently, the MTT solution (0.5 mg/mL), which was diluted in culture medium without fetal bovine serum (FBS), was added to the wells, and the plates were incubated for 2 h. After the incubation, the MTT solution was removed, and 100 µL of DMSO was added to each well. Finally, the absorbance was measured at 540 nm using an Elisa ELx800 microplate reader (BioTek Instruments, Inc., Winooski, VT, USA) with the SoftMax Pro software version 5.4.1.

### 4.6. Cell Proliferation Assay

Cell proliferation was assessed by the direct counting of nuclei using DAPI (4′,6-diamidino-2-phenylindole) for nuclear staining (Molecular Probes, Eugene, OR, USA). RAECs (2.5 × 10^3^) and ARPE-19 cells (1 × 10^4^) were seeded in 96-well plates and cultured for 18 h in 10% FBS. They were then treated with different concentrations of 6-O-desulfated heparin (0.001 mg/mL, 0.01 mg/mL, or 0.1 mg/dL) in complete culture medium. At 24 and 48 h post-treatment, the cells were washed with PBS and fixed with methanol for 20 min. Subsequently, cells were washed with PBS and stained with DAPI diluted 1:10,000 for 1 h in the dark. The cells were then washed with PBS and analyzed using the Incell Analyzer 2200 (GE Healthcare Bio-Sciences Corp., Uppsala, Sweden).

### 4.7. Capillary Tube Formation Assay

For this assay, 70 µL of Matrigel^®^ (Corning, New York, NY, USA) was plated per well in a 96-well plate [21]. After a polymerization period of 4 h of incubation (37 °C, 2.5% CO_2_), 4 × 10^4^ RAECs were plated on top of the Matrigel and treated with different concentrations of Hep-6Od (0.001 mg/mL, 0.01 mg/mL, or 0.1 mg/mL). Subsequently, the cultures were maintained at 37 °C in a 2.5% CO_2_ atmosphere for 16 h. Tube formation was examined under an inverted bright-field microscope (Zeiss Primovert, Carl Zeiss Microscopy, LLC., Jena, Germany) at 40× magnification. The total length of tubular structures in Matrigel was measured and determined using ImageJ analysis software (version 1.54p, angiogenesis plugin) (NIH, Bethesda, MD, USA) and expressed as tube length in millimeters (mm) [21].

### 4.8. Cell Migration Assay (Wound Healing Assay)

The wound healing assay was conducted to evaluate cell migration, simulating in vitro healing processes [32]. RAECs were seeded in a 24-well plate at a density of 5 × 10^4^ cells/well in F12 culture medium and cultured in a 2.5% CO_2_ atmosphere at 37 °C. Upon reaching confluence, wounds were created using a 200 µL micropipette tip in each well, followed by the addition of different concentrations of Hep-6Od (0.1, 0.01, or 0.001 mg/mL) or a control (PBS). Sequential images were taken at 0, 24, 48, and 72 h post-injury using a bright-field microscope (Zeiss Primovert, Carl Zeiss Microscopy, LLC., Jena, Germany) at 100× magnification. The area of wound closure (μm^2^) was quantified using ImageJ software (NIH, Bethesda, MD, USA).

### 4.9. Molecular Interaction Assay: 6-O-Desulfated Heparin and Growth Factors (FGF-2)

To assess the possible interaction between FGF-2 and both unfractionated heparin (UFH) and Hep-6Od, a protein thermal stability assay was performed using differential scanning fluorimetry (DSF). FGF-2 (0.2 mg/mL) (Thermo Fisher Scientific Inc., Waltham, MA, USA(, UFH (1 mg/mL), or 6-O-desulfated heparin (10 mg/mL) were mixed with SYPRO^®^ Orange protein gel stain (Invitrogen, Carlsbad, CA, USA) that was previously diluted in PBS (1:50) to achieve a final volume of 35 µL. These solutions were then dispensed into a 96-well optical plate, with 10 µL per well across six wells for each solution. FGF-2 without ligands served as a control. The plate was sealed with optical adhesive film from Applied Biosystems, Waltham, MA, USA, with a 2 min preheating phase at 32 °C, followed by a temperature gradient ramping from 32 °C to 99.9 °C. During this phase, the temperature increased by 0.7 °C per cycle, with each cycle lasting 5 s. Data collection was performed every 5 s with the Applied Biosystems 7500 Real-Time PCR system. The fluorescence versus temperature curves were processed by calculating their first derivatives and processed using GraphPad Prism 7.0 (GraphPad Software, La Jolla, CA, USA).

### 4.10. In Vivo Experiment

The in vivo study received ethical approval from the Federal University of São Paulo’s Ethics Committee on Animal Use (CEUA/UNIFESP, protocol no. 990022081) and was conducted at the Center for the Development of Experimental Models for Biology and Medicine (CEDEME) at UNIFESP, in accordance with the guidelines of the Association for Research in Vision and Ophthalmology (ARVO).

Sixteen male isogenic lean Zucker rats (aged 8–12 weeks, weighing 180–250 g) were used and randomly allocated into four groups, with four animals per group. The rats were housed under a 12:12 h light–dark cycle at a constant room temperature, with ad libitum access to water and standard chow.

#### 4.10.1. Induction of Choroidal Neovascularization

Anesthesia was induced by an intraperitoneal injection of a mixture of ketamine (10%) and xylazine (2%) (Syntec do Brasil Ltda, Santana do Parnaíba, São Paulo, Brazil) in an 8:2 ratio (80 μL of ketamine to 20 μL of xylazine). A volume of 0.1 mL of this anesthetic solution was administered per 100 g of body weight. Pupil dilation and topical anesthesia were achieved using tropicamide (10 mg/mL) and phenylephrine (2.5%) (Allergan, Guarulhos, São Paulo, Brazil).

For the laser procedure, each animal was positioned in front of a slit lamp, with a microscope slide coated with 2% methylcellulose serving as a contact lens substitute. Only one eye per animal was subjected to the experimental procedure.

Choroidal neovascularization (CNV) was induced using a green argon laser (Quantel Medical, Cournon-d’Auvergne, France; 532 nm wavelength) set at a power of 100–120 mW with a spot size of approximately 100 microns and a duration of 100 milliseconds per shot. Four laser burns were applied around the optic disc in each treated eye, with the rupture of Bruch’s membrane confirmed by the appearance of a subretinal whitish air bubble [21].

#### 4.10.2. Intravitreal Injection of 6-O-Desulfated Heparin (6-OHep) and Control Treatment

Following Bruch’s membrane rupture, 6-O-desulfated heparin (3 μL) or BSS (for control) was intravitreally injected using a micro syringe (Hamilton Co, Reno, NV, USA). The animals received 0.05 µg, 0.5 µg, or 5 µg of Hep-6Od (in distilled water) or 5 µL of the BSS control solution (Alcon, São Paulo, Brazil).

To calculate the intravitreal concentrations of Hep-6Od in vivo, we assumed an average vitreous volume of 50 μL in Zucker rats, as previously reported [27,28] The same concentration range (10–100 μg/mL) was then applied to endothelial cell cultures in vitro to ensure consistency and physiological relevance between the experimental models.

Injections were precisely administered using a Stemi 508 stereo microscope (Carl Zeiss, Oberkochen, Germany) to ensure correct drug delivery into the vitreous cavity.

The following day, animals underwent fundoscopic examinations to identify and exclude any with traumatic lens damage or vitreous hemorrhage [21].

#### 4.10.3. Euthanasia, Enucleation, and Immunofluorescence Analysis

Fourteen days after the intravitreal injection, the animals were euthanized by the intraperitoneal administration of an anesthetic cocktail consisting of 250 µL of 10% ketamine hydrochloride and 250 µL of 2% xylazine hydrochloride (both from Syntec do Brasil Ltd.a, Santana do Parnaíba, São Paulo, Brazil), followed by an additional 100 µL of the same mixture to induce cardiopulmonary arrest. One eye per rat was immediately enucleated and fixed with 200 µL of 4% paraformaldehyde for 2 h at room temperature.

Subsequently, the eyes were rinsed with PBS and stored overnight at 4 °C in 20% sucrose (Sigma-Aldrich, St. Louis, MO, USA). After an additional PBS rinse, the eyecups were dissected, flat-mounted, and permeabilized using a blocking solution containing 2% bovine serum albumin (BSA) and 0.1% saponin in PBS.

The following day, the eyecups were incubated with an anti-von Willebrand factor primary antibody (sc-8068, Santa Cruz Biotechnology, Dallas, TX, USA) diluted 1:50 in PBS with 1% BSA, and maintained overnight at 4 °C. Subsequently, they were incubated with an Alexa Fluor 488-conjugated secondary antibody (Thermo Fisher Scientific Inc., Waltham, MA, USA) at a 1:300 dilution for 30 min at room temperature, protected from light.

For the choroidal neovascularization analysis, choroidal flat mounts were immunostained for von Willebrand factor (vWF), a marker constitutively expressed throughout the native choroidal vasculature. The eyecups were mounted onto microscope slides using Fluoromount-G mounting medium (Electron Microscopy Sciences, Hatfield, PA, USA) for the lesion analysis. To distinguish neovascular lesions from physiological vWF expression, images were taken using an Axio Observer Z1 inverted fluorescence microscope (Zeiss, Oberkochen, Germany) and the laser intensity was normalized using choroidal areas that had not received laser shots, allowing the visualization exclusively of regions with pathologically increased vWF fluorescence. These areas of focal hyperfluorescence, corresponding to neovascularization, were located in the quadrants where the laser spots had been applied.

Measurements of lesion areas (in µm^2^) were performed using ImageJ software (National Institutes of Health, Bethesda, MD, USA). The hyperfluorescent circular lesions were manually delineated and analyzed with this software and the neovascularized area was calculated based on the defined perimeter/area. The immunofluorescence analysis focused on areas indicative of laser-induced disruption of Bruch’s membrane, while hemorrhagic regions were excluded from quantification [21].

### 4.11. Statistical Analysis

The evaluation of significant differences was performed using one-way ANOVA complemented by the Bonferroni correction for multiple comparisons utilizing the software GraphPad Prism 7.0 (La Jolla, CA, USA). The results are expressed as the mean values ± the standard errors. The analysis was framed within a 95% confidence level, setting the alpha for significance at 0.05. Therefore, outcomes yielding *p* values below 0.05 were acknowledged as statistically significant.

## Figures and Tables

**Table 1:** Melting temperature (Tm) values in °C corresponding to the peak of the first derivative of each melting curve. The table presents the Tm values of FGF2 without a ligand and when associated with unfractionated heparin (UFH) and 6-O-desulfated heparin (Hep6OH).

	Tm °C
FGF-2	51.95
FGF-2 + UFH	82.0
FGF-2 + Hep6-Od	85.5

## Data Availability

The data presented in this study are available upon request from the corresponding author.

## Abbreviations

The following abbreviations are used in this manuscript:

Hep-6oD	6-O-Desulfated Heparin
UFH	Unfractionated Heparin
RAECs	Rabbit Aortic Endothelial Cells
ARPE-19	Adult Retinal Pigment Epithelium 19
CNV	Choroidal Neovascularization
